# Editorial: Development and plasticity of multisensory circuits

**DOI:** 10.3389/fncir.2022.1129196

**Published:** 2023-01-13

**Authors:** Jason W. Triplett, Benjamin A. Rowland, Michael Reber

**Affiliations:** ^1^Center for Neuroscience Research, Children's National Hospital, Washington, DC, United States; ^2^Department of Pediatrics, The George Washington University School of Medicine and Health Sciences, Washington, DC, United States; ^3^Department of Neurobiology and Anatomy, Wake Forest University School of Medicine, Winston-Salem, NC, United States; ^4^Donald K. Johnson Eye Institute, Krembil Research Institute, University Health Network, Toronto, ON, Canada

**Keywords:** brainstem, inferior colliculus, spontaneous activity, computational modeling, visual-somatosensory integration

Different attributes of the external environment are initially detected and processed by distinct sensory systems and later integrated in multisensory areas of the brain. Multisensory integration is critical for establishing a comprehensive percept of the world, detecting salient stimuli, and communicating effectively. Indeed, the prevalence of deficits in sensory and multisensory processing in multiple neurodevelopmental disorders underscores the importance of establishing the precise patterns of connectivity needed for such complex computations. Nature and nurture work together to instruct the wiring utilized in multisensory integration. Converging unisensory inputs are initially guided by genetically regulated molecular and activity-dependent mechanisms to align neuronal inputs relaying related signals (e.g., signals derived from the same areas of space) (Triplett et al., 2009; Tikidji-Hamburyan et al., 2016; Savier et al., 2017, 2020). These connections are later reshaped through experience-dependent plasticity that modifies multisensory processing rules of inter-sensory competition and cooperation (Yu et al., 2013; Murray et al., 2016). These changes can continue well into adulthood. Remarkably, the principles underlying multisensory circuit development and plasticity have begun to be leveraged to ameliorate sensory deficits (Legge and Chung, 2016; Daibert-Nido et al., 2021). However, several gaps remain in our understanding of the mechanisms underlying each of these stages, hampering the development of more effective therapies. In this Research Topic we bring together exciting new research and relevant reviews regarding the development, organization, and plasticity of multisensory systems at multiple levels.

To optimize efficient processing, neuronal connections of sensory neurons are highly organized to reflect the types of information they detect, as highlighted by Fritzsch et al. regarding somatosensory, vestibular, auditory, and gustatory projections from the brainstem. There, somatosensory and auditory information are organized into continuous topological maps, in which neighboring neurons are responsive to adjacent parts of the body (somatosensory) or tonal frequencies (auditory). In contrast, vestibular and gustatory information is projected to the brain in a discrete manner, divided into regions tuned to distinct movement directions or tastes. Ultimately, these diverse senses are integrated in telencephalic regions of the brain by neurons that receive unisensory inputs as well as tertiary inputs relaying context-dependent information.

Intriguingly, discrete organization can also be observed in multisensory integration centers that receive sensory inputs typically organized in a continuous manner. In the lateral cortex of the inferior colliculus (LCIC) somatosensory and auditory information converges, however, projections are maintained in distinct unimodal patches. It remains unclear how this remarkable organization develops. Weakley et al. have now elucidated the timing and trajectory of somatosensory patch formation in the LCIC, providing a framework to begin dissecting mechanism. Initially diffuse projections from primary somatosensory cortex at postnatal day 0 (P0) are rapidly sorted into distinct patches by P4 and adult-like by P12. This timing is concurrent with that of auditory patch formation and overlaps with both the expression of critical molecular cues and the onset of audition (Lamb-Echegaray et al., 2019). Experiments manipulating each independently or both together are needed to tease apart the relative contributions of nature and nurture in this multisensory circuit.

While the mechanisms underlying circuit formation in the LCIC remain unclear, significant advances have been made into the interactions between neuronal activity and molecular signaling utilized to shape circuit connectivity. In many sensory systems, two distinct forms of activity play critical roles in circuit formation: spontaneously generated and sensory evoked. Spontaneous activity is typically highly structured. For example, in the visual system, waves of activity in which local clusters of retinal ganglion cells are active propagate across the retina prior to visual experience. In contrast, visually-evoked activity is much less structured, given the diverse array of visual scenes encountered by an organism. Each of these forms of activity play critical, but distinct, roles in circuit development, but how can a neuron distinguish between them? An elegant review by Pumo et al. highlights differences in chromatin modifications at immediate early gene and late responsive genes as at least one mechanism that distinct transcriptional programs are induced by spontaneous vs. sensory-evoked activity. While more needs to be understood regarding the transcriptional programs and signaling pathways invoked in the context of multisensory plasticity, the field is clearly making significant advances.

To further understanding of the mechanisms of plasticity and underlying computations utilized by multisensory circuits, two papers in this collection adopted computation modeling approaches. In the first, Shaikh approaches the intriguing hypothesis that as multisensory circuits develop, changes in synaptic weight reflect the reliability of sensory cues. To do so, an experience-naïve simulated robot was tasked with localizing a target that produced visual and auditory cues, the reliability of which could be changed independently and systematically. Random trials showed that the multisensory computation was weighted proportional to the relative reliabilities of each stimulus, as predicted by a Bayesian framework. There was also a bias toward the visual modality, which was consistent with human perceptual studies. This provides a conceptual and quantitative framework within which to appreciate the development of multisensory integration as the product of a statistical learning process. These results will help guide the design of future empirical studies and stimulate examinations of the differences and commonalities between multisensory processes and other domains of statistical learning. The second computational study sought to elucidate the mechanisms underlying an interesting phenomenon in which the effect of a visual distractor on somatosensory localization persists after removal of the distractor cue (Cuppini et al.). Many models of plasticity describe changes that take place over long periods of time and specify singular slow-evolving mechanisms, but different mechanisms are required for the brain to make rapid adaptations. Here, Cuppini et al. use a neurocomputational model to describe how the visual-somatosensory interaction and perceptual aftereffect can be produced by rapid changes in multiple circuits. This represents an important contribution in furthering our understanding of how simultaneous changes spanning multiple areas collectively shape both normal and anomalous perception.

In conclusion, this is an exciting time in the field of multisensory circuit biology. Combining experimental and theoretical approaches, researchers are bringing to bear an array of conceptual frameworks and techniques to interrogate the development, organization, function, and plasticity of networks capable of efficient processing of complex data. Elucidating the mechanisms of multisensory integration at multiple levels is not only a rewarding task for neuroscience, but also can help identify circuit changes underlying deficits in, and hopefully therapies to treat, neurodevelopmental disorders and other disorders of sensory processing.

## Author contributions

JT, BR, and MR wrote and edited the manuscript. All authors contributed to the article and approved the submitted version.
